# Biobanks as an Indispensable Tool in the “Era” of Precision Medicine: Key Role in the Management of Complex Diseases, Such as Melanoma

**DOI:** 10.3390/jpm14070731

**Published:** 2024-07-06

**Authors:** Alessandro Valenti, Italia Falcone, Fabio Valenti, Elena Ricciardi, Simona Di Martino, Maria Teresa Maccallini, Marianna Cerro, Flora Desiderio, Ludovica Miseo, Michelangelo Russillo, Antonino Guerrisi

**Affiliations:** 1 Radiology and Diagnostic Imaging Unit, Department of Clinical and Dermatological Research, San Gallicano Dermatological Institute IRCCS, 00144 Rome, Italy; flora.desiderio@ifo.it (F.D.); ludovica.miseo@ifo.it (L.M.); antonino.guerrisi@ifo.it (A.G.); 2 SAFU, Department of Research, Advanced Diagnostics, and Technological Innovation, IRCCS-Regina Elena National Cancer Institute, 00144 Rome, Italy; italia.falcone@ifo.it; 3 UOC Oncological Translational Research, IRCCS-Regina Elena National Cancer Institute, 00144 Rome, Italy; fabio.valenti@ifo.it (F.V.); elena.ricciardi@ifo.it (E.R.); 4 UOC Pathology Unit, Biobank IRCCS-Regina Elena National Cancer Institute, 00144 Rome, Italy; simona.dimartino@ifo.it; 5 Department of Clinical and Molecular Medicine, Università La Sapienza di Roma, 00185 Rome, Italy; mariateresa.maccallini@ifo.it (M.T.M.); marianna.cerro@ifo.it (M.C.); 6 Division of Medical Oncology, IRCCS-Regina Elena National Cancer Institute, 00144 Rome, Italy; michelangelo.russillo@ifo.it

**Keywords:** biobanks, precision medicine, melanoma, tissue biobank, liquid biobank, digital biobank, imaging biobank

## Abstract

In recent years, medicine has undergone profound changes, strongly entering a new phase defined as the “era of precision medicine”. In this context, patient clinical management involves various scientific approaches that allow for a comprehensive pathology evaluation: from preventive processes (where applicable) to genetic and diagnostic studies. In this scenario, biobanks play an important role and, over the years, have gained increasing prestige, moving from small deposits to large collections of samples of various natures. Disease-oriented biobanks are rapidly developing as they provide useful information for the management of complex diseases, such as melanoma. Indeed, melanoma, given its highly heterogeneous characteristics, is one of the oncologic diseases with the greatest clinical and therapeutic management complexity. So, the possibility of extrapolating tissue, genetic and imaging data from dedicated biobanks could result in more selective study approaches. In this review, we specifically analyze the several biobank types to evaluate their role in technology development, patient monitoring and research of new biomarkers, especially in the melanoma context.

## 1. Introduction

Since the initial institution, biobanks have been given many definitions that could well delineate their functions. Probably, the Pan-European Biobank and Biomolecular Resources Research Infrastructure (BBMRI) gave the best definition of biobanks as repositories of biological samples and associated information needed for biotechnological advancement, public health and research [1]. Nowadays, the primary aim of personalized medicine is not to find a universal cure but rather the optimal one for each unique patient. This suggests that a deep knowledge of the disease in its various aspects and the ability to stratify patients as much as possible according to common characteristics are essential processes. Biobanks allow for a massive quantity of biological and non-biological materials obtained from great patient populations to be catalogued in a homogeneous and structured manner. This allows for a 360-degree view of pathology by exploring its genetic, phenotypic and molecular aspects [2,3]. In recent decades, biobanks are in an age of high development, since the significant expansion of translational studies requires an increasingly impressive number of biological materials, imaging and related information [4,5]. Unlike what happened in the past, biobanks do not only play the main role of collecting patient samples and/or data but also implement their operations according to standardized procedures and rigid protocols necessary for obtaining reliable and reproducible samples everywhere [6]. The evolution of personalized medicine and the development of omics sciences have resulted in the production of massive quantities of data (big data) associated with the individual disease and, more importantly, the individual patient. Within a broad vision, the cataloging of these data by classical and digital biobanks is, therefore, crucial. Indeed, high quality and orderly accessible samples will allow for the development of more targeted lines of research for the improvement of patient treatments [7,8]. In 2018, an interesting study conducted in China found that hospitals with big biobanks exhibited a scientific and technological output and academic impact of 48.35%, 55.16% and 58.65% higher, respectively, than hospitals with the same characteristics but small biobanks [9]. This result appears extremely important and significant because it highlights the fundamental role of biobanks and their organization in the various stages of scientific processing. The integration of genomic, tissue and imaging data, stored and managed in dedicated biobanks, could be a key breakthrough in the management of complex diseases. Melanoma could benefit by this approach, as it represents a heterogeneous disease with multiple genetic and molecular variants that influence its progression and treatment response. The integration of different data would allow for the development of the large-scale studies necessary for the understanding of still unclear aspects related to melanoma progression, metastases and refractory treatments [10,11].

## 2. Biobanks: General Aspects

Biobanks represent collections of biological and computational materials and digital images of large patient populations and are responsible for storing, cataloging and preserving personal information, such as medical records, family histories and genetic information, for research aims [3]. Depending on the nature of the material collected, preservation techniques and scope of research, biobanks are a highly heterogeneous reality. To date, a first classification involves “population-based” and “disease-oriented” biobanks. The first ones link molecular data and genetic predispositions of a general population to the development of specific diseases; the second ones, on the other hand, focus their activities on collecting disease-specific samples [8,12]. Additional approaches for classifying biobanks are based on the type of research they intend to support and the types of samples collected, such as tissue, DNA/RNA, blood, etc. [13,14]. Lastly, another category is virtual biobanks, which are electronic archives of biological samples and other related data that are independent and separate from the places where samples are really and physically stored [12,15].

In addition to their primary archival purpose, biobanks (beyond their nature) also arise to standardize procedures for the collection, storage and quality control of banked samples. Integrity in the preservation and quality of samples (combined with their associated biological and digital data), in an era of precision medicine, appears necessary for the development of more complex and large-scale research. For this purpose, in recent years, multi-institutional projects aimed at standardizing biobank procedures have proliferated to ensure that unequivocal and comparable materials can be used by anyone anywhere [3]. In the future, biobanks will need continuous updates to maintain high levels of standardization. The use of new technologies for the management and maintenance of biological and nonbiological data will need to be implemented, and genetic and clinical information will need to be constantly checked and renewed.

### 2.1. Ethical and Legal Regulation

The use of biological samples and the massive sharing of databases containing genotypic/phenotypic data have generated important ethical questions about the use and disclosure of information closely related to the individual. In this regard, precise and astringent regulations have been developed to protect the privacy and intent of the individual patient/donor. In 1964, the Helsinki Declaration established the basis for regulating human medical research by moving toward more controlled studies. In this sense, that declaration stipulated that any new study on humans should be examined by an independent committee and based on objective results obtained with laboratory animals [16]. Over the years, even following the development of more advanced and sensitive technologies, it has been necessary to implement the regulation related to biomedical research that, to date, must have an informed consent (IC) by the patient. Although there is no international consensus, the purpose of the IC is to enable an individual to decide whether to participate in a research program. All biobanks have delineated a “broad-spectrum” IC to include within it all research purposes related to the biobanking of materials or information. Generally, the ICs of biobanks are highly detailed, but there are common points such as the following [17]:

✧ Description of the materials to be biobanked;
✧ Principles of data sharing with other institutions;
✧ Principles for ‘researchers’ access to data;
✧ Privacy regulations;
✧ Possibility of withdrawal from the study;
✧ Dispositions in case of death;
✧ Principles of non-profit.

### 2.2. Privacy Policy

A finely regulated aspect of biobank management is the respect of the privacy of all participants. Essentially, personal data are considered private information because they could be personally harmful and a source of discrimination (e.g., information related to HIV or other diseases). Biological or non-biological samples present at all biobanks can be of four types, characterized by a different privacy level. Anonymous samples benefit from the maximum privacy level, as they are not associated with any identity. They are generally collected during screening activities. Anonymized samples, on the other hand, are obtained from identified subjects but suffer a process of anonymization before being used for any purpose. In some circumstances, samples originate from known individuals whose associated data are made available and used through coded information; in this case, the samples are defined as identifiable. Lastly, samples obtained from known individuals whose data are exposed are termed identified [18].

## 3. Biobank Types

Early biobank types essentially started for the controlled storage of biological materials destined for research or therapeutic purposes. However, with the advancement of new technologies and precision medicine, it naturally became necessary to expand the biobank concept to very varied types of samples. Obviously, different workflows can be differentiated in the various types of biobanks, all of which, however, flow toward the correct and standardized storage of pertinent materials.

### 3.1. Tissue Biobank

**Figure 1:**
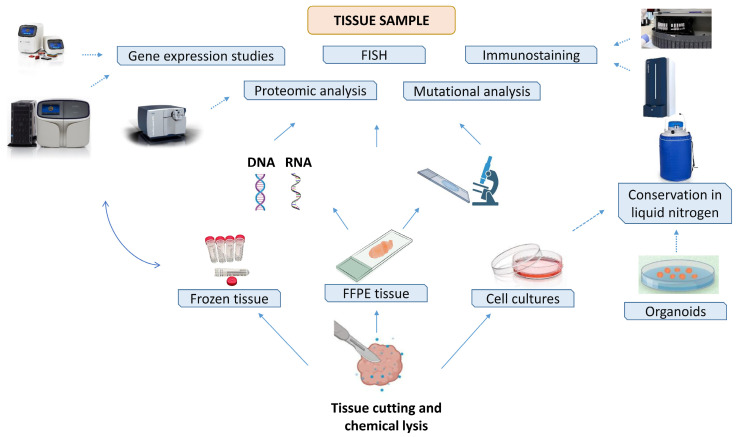
Schematic workflow in a tissue biobank.

Tissue biobanks are dedicated to the collection of all tissues, especially resulting from surgical retrievals, which allow for the best analysis of a specific pathology. Although paraffin-preserved tissues still dominate, frozen tissue turns out to be the perfect biological sample for the most advanced analytical methods that need nucleic acids and proteins of better quality and yield [19]. Although the collection of frozen samples still appears to be for almost exclusive use by research programs, they will soon be “bread and butter” for biobanks. Indeed, in recent years, increasingly reliable and accurate sequencing methods are entering the clinical scenario in a strong manner, thus prompting the management of an increasing number of frozen samples [4]. Particularly in oncology, where an extensive study of the tumor is necessary for the clinical management of the patient, tissues are processed to obtain cell lines, organoids, DNA and RNA. All of this information, indeed, will allow for a deeper analysis of each individual tumor and will point toward precision medicine. Both frozen and formalin-fixed and paraffin-embedded tissues present benefits and limitations [20,21]. Paraffin-embedded tissues, albeit easier to manage because they are stored at room temperature in nonspecific storage facilities, do not always allow for the acquisition of materials of adequate quality, especially for more advanced sequencing techniques [21]. Samples stored at ultra-low temperatures can potentially be archived for decades, preserving a high quality of DNA and RNA and even protein enzyme activity. However, not all biobanks have the necessary personnel or infrastructure to store frozen biological samples. In addition, the preservation of these samples also needs very high-quality standards because several pathogens are able to survive at low temperatures [4] (Figure 1).

### 3.2. Cell and Organoid Biobank

A large part of scientific research, in particular oncology research, involves the use of cell systems needed for initial drug-screening and/or molecular profiling analyses. Frequently, the cell lines used in various laboratories may not match or may be incorrectly identified. Therefore, it has been necessary to establish cell biobanks to provide users with standardized and reliable information on the origin of each line, correct expansion procedures and maintain cultures (Figure 1). Not surprisingly, the scientific community requires the acceptance of scientific work and periodic authentication of the cell lines used by certified institutions, such as the American Type Culture Collection (https://www.atcc.org, accessed on 1 July 2024), Leibniz Institute DSMZ-German Collection of Microorganisms (https://www.dsmz.de, accessed on 1 July 2024), European Collection of Authenticated Cell Cultures (https://www.culturecollections.org.uk/about-us/ecacc, accessed on 1 July 2024) and others. In addition, the establishment of tissue biobanks at hospitals and research institutes has implemented the possibility of producing new cell lines directly from patients’ surgical pieces [22]. Also, in this case, the standardization of isolation processes and appropriate biobanking are necessary for obtaining valid and reproducible scientific results.

Cell lines, although isolated from patients, are a very simplified model that cannot reflect the entire characteristics and components of the original tissue. Thus, while optimal for early study phases, they need the support of other, more complex approaches. In recent years, scientific research has been focusing on complex three-dimensional, multicellular structures, defined as “organoids”, that recapitulate the original tissue after insertion into a 3D matrix [23] (Figure 1). Particularly in oncology, this new approach is revolutionary because it permits an organized structure that retains most of the components of the tissue to be reproduced on the plate. Clearly, this results in a more complete view of the tumor and the possibility of more personalized analysis and screening. Of course, the use of organoids is not only limited to cancer research, but they can be implicated for regenerative medicine, such as that focused on organ development, and new drug testing [24,25]. In view of this great scientific potential, a number of organoid biobanks are being implemented [26,27,28,29,30]. In parallel with the great potential, unlike other biobanks, those of organoids have important limitations. These are essentially related to a still poor standardization of both isolation and preservation processes connected with the high heterogeneity of the origin tissues. In addition, it is still very complex to maintain all tumor heterogeneity in the organoid because part of the immune system is lost early on [31].

### 3.3. Liquid Biobank

**Figure 2:**
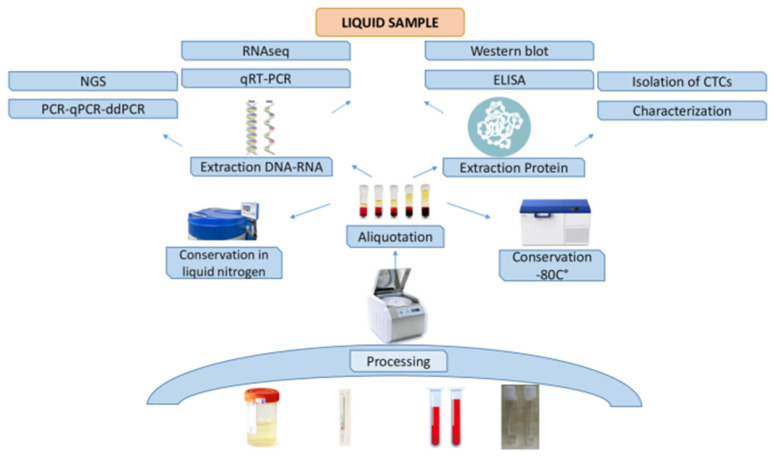
Schematic workflow of a liquid biobank.

Blood represents an inestimable source of information, and the study of its components and their variations is commonly used in basic and translational scientific research. Especially in oncology, blood (but also other fluids, such as urine, saliva and cerebrospinal fluid) is emerging as a valuable and alternative approach to understanding the tumor. In blood, indeed, circulating tumor cells and their genetic material, released as a result of their death or cell division, are present [32]. So, their study and monitoring over time can provide precious information to clinicians for disease clinical management and are fertile ground for the research of new tumor biomarkers [32]. For this reason, in recent years, tissue biobanks have been supported by liquid biobanks, constituting an invaluable source of scientific information (Figure 2).

Depending on the needs and tests to be performed, blood may be collected in its entirety or divided into plasma and serum. Conservatives and additives in the tubes used for blood collection may vary according to the specific application [33,34]. Serum is necessary in some biochemical tests, while tests based on DNA and/or RNA analysis require coagulated blood (including plasma, buffy coat and red blood cells). For whole-blood samples, 6 mL labeled vacutainer tubes with the anti-coagulant Na_2_-EDTA are used, whereas for blood serum, 6 mL labeled vacutainer tubes containing a thixotropic barrier gel at the bottom of the tube are preferred [35]. All blood components are easily degraded, and therefore, their processing and storage require a short time. The maximum time between sample collection and processing depends on the component to be analyzed [33]. Generally, samples should be maintained at 4 °C, and an interval of 24 h between collection and processing is considered an acceptable time [36] (Figure 2).

### 3.4. Imaging Biobank

**Figure 3:**
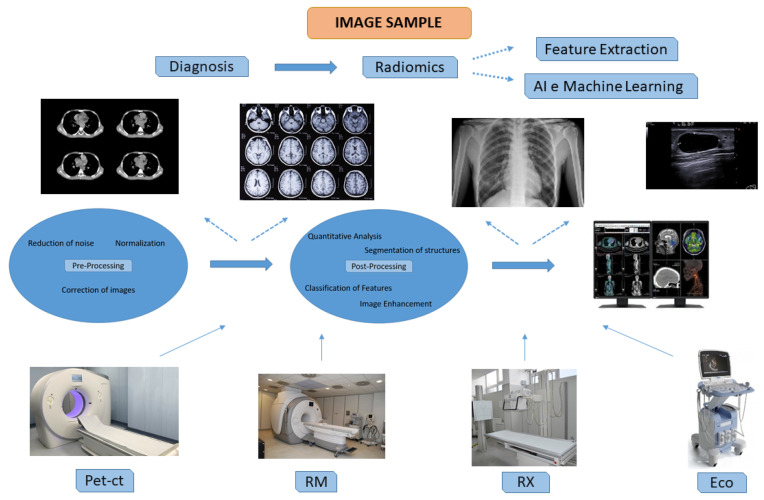
Process analysis of image samples.

The development of ever more sensitive diagnostic imaging technologies and the onset of artificial intelligence (AI) have allowed for a new study approach: radiomics. This approach, although not yet clinically validated, is powerfully emerging, especially in the study of many cancer forms, because, by extracting tumor-specific quantitative features from biomedical radiological images, it can help in treatment optimization and patient management [10,37]. Specifically, radiomics, in combination with tumor texture analysis, may provide information on new imaging biomarkers to associate with already known molecular biomarkers [37]. Obviously, these methodological approaches require technological training and a massive amount of digital data. For this reason, the creation of imaging biobanks is essential. Imaging biobanks are virtual infrastructures with massive storage capacities and, therefore, require standardization and validation procedures for the elaborated images [38] (Figure 3).

The European Society of Radiology (ESR) defined an imaging biobank as medical and biomarker imaging shared among multiple researchers [38]. An example is represented by the Imaging Data Commons (IDC) that provides open access to oncology imaging collections supported by patient’s clinical data (https://portal.imaging.datacommons.cancer.gov, accessed on 1 July 2024). The IDC follows FAIR (findable, accessible, interoperable, reusable) principles to ensure the interoperability and consistency of the data. All data are harmonized in the DICOM format to promote search standardization and analysis [39]. High-performance standard infrastructures, such as the Minimum Information About Biobank Data Sharing Initiative (MIABIS) (https://www.bbmri-eric.eu/howtomiabis, accessed on 1 July 2024) and Observational Medical Outcomes Partnership (OMOP) Common Data Model (CDM) (https://www.ohdsi.org/data-standardization, accessed on 1 July 2024) facilitate the exchange and systematic analysis of data, ensuring traceability, accessibility and interoperability following the FAIR principles [40].

Although not yet as developed as the biobanks of biological samples, the imaging ones, in recent years, are known to have a very prosperous era of development, and the evidence lies in the several projects that are being developed [40]. For example, a massive British study, started in 2006, biobanked imaging data on the brains, hearts, abdominal composition, bones, joints and blood vessels of 100,000 participants. This has resulted in a wealth of information that, linked to phenotypic and genetic sources, is an indispensable resource for solving current unanswered clinical questions [41]. In oncology, an Italian project (NAVIGATOR) aims at the development of an imaging biobank aimed at collecting and storing a large amount of standardized multimodal imaging datasets, including computed tomography data, MRI and positron emission tomography, with patient information and omic analysis. Data from patients with prostate, rectal and gastric cancer will be used to create a digital model of the patient, supporting reliable prediction of the disease phenotype and risk stratification [42]. Additional international projects aim to develop artificial intelligence platforms that can integrate data and imaging models to fuel and support precision medicine [43,44]. As described above, image biobanks find fertile ground, especially in the tumor field, because cancer patients often carry out serial controls (with different imaging protocols) [40]. Patients with metastatic melanoma, for example, are subjected to radiological controls periodically during the disease follow-up and for the monitoring of target therapies and immunotherapy response.

### 3.5. Digital Biobank

The large quantity of biomedical data of a genetic, imaging and diagnostic nature is an invaluable source of information on which scientific research can draw. This is, however, only possible if these data can be easily accessed by all in a standardized and controlled manner. Digital biobanks are created precisely for the purpose of facilitating the sharing of data of various types necessary for the development of a personalized approach and management of individual disease. The digital biobank is a supporting infrastructure in which the integration of various omics data (genomic, proteomic, metabolomic, radiomic) is possible for a disease-comprehensive approach [8]. Although digital biobanks have enormous potential, to date, they are still poorly developed, and approaches on how to implement their use in translational medicine are still lacking. One reason lies in the need for cooperation among the different professionals involved (biologists, pathologists, clinicians, informaticians) and the development of new advanced algorithms and computer-aided techniques.

Examples of digital biobanks are the following:

1. Cancer Genome Atlas (TCGA) is a digital biobank focused on oncological research that provides public access to an extensive catalog of genomic and epigenomic data (https://portal.gdc.cancer.gov, accessed on 1 July 2024) [45];
2. The European Genome and Phenome Archive (EGA) is a digital biobank focused on storing genetic, phenotypic and clinical data from different research projects while maintaining control over access to data at the sender level (https://ega-archive.org, accessed on 1 July 2024) [46];
3. The Global Alliance for Genomics and Health (GA4GH) is an advanced digital biobank that addresses the challenges of the growing production of sequencing data from diverse study populations (https://www.ga4gh.org, accessed on 1 July 2024) [47];
4. U.K. Biobank aims to collect, store and manage information and samples from 5,000,000 participants to enable genetic and nongenetic investigations of diseases of aging. It is among the most efficient digital entities and enables sharing through online platforms and systems (https://www.ukbiobank.ac.uk, accessed on 1 July 2024) [48].

## 4. Biobank Sustainability

Biobanks (biological and digital) need constant funding for their sustainability. Over the past decades, the exponential increase in biobank data has implemented costs related to management, maintenance and involvement of dedicated staff. Often, due to their non-profit nature, the financial resources of biobanks are linked to public funding for research [3,49]. Obviously, this kind of financial approach cannot be “long-term” but is closely related to the funding period, which, in most cases, fails to cover all costs related to sample collection, sample processing, management and distribution and all expenses related to infrastructure and administration. In more than a few cases, biobanks have had to stop their activities due to a lack of appropriate funding. A striking example was the failure of the Singapore biobank, established in 2002, that was forced to close a few years later due to insufficient government aid in the face of high operating costs [49]. One of the most influential aspects of biobank finance is the cost of materials, energy and space dedicated to the physical storage of biological and digital samples. In this regard, meetings of the International Society for Biological and Environmental Repositories (ISBER) (https://www.isber.org/, accessed on 1 July 2024) and the European Middle Eastern and African Society for Biopreservation and Biobanking (ESBB) (https://esbb.org, accessed on 1 July 2024), focused on cost management related to the energy consumption, waste materials, circular economy and economic sustainability of biobanks, have been held punctually in recent years [50].

Biobank sustainability is closely connected not only with its ability to self-manage available finances but also to raise new funds. This is only possible if the biobank is able to establish external scientific collaborations by contributing with standardized data [51]. Access to quality samples and data and high project completion rates are critical to the success of a biobank. Having a well-structured project allows for biobanks to provide critical resources for global scientific research, ensuring safety, sample quality and the input needed, so that the data do not remain confined to its own biobank but are the subject of collaboration and studies on multiple fronts.

## 5. Melanoma Precision Medicine: Biobanking Role

**Figure 4:**
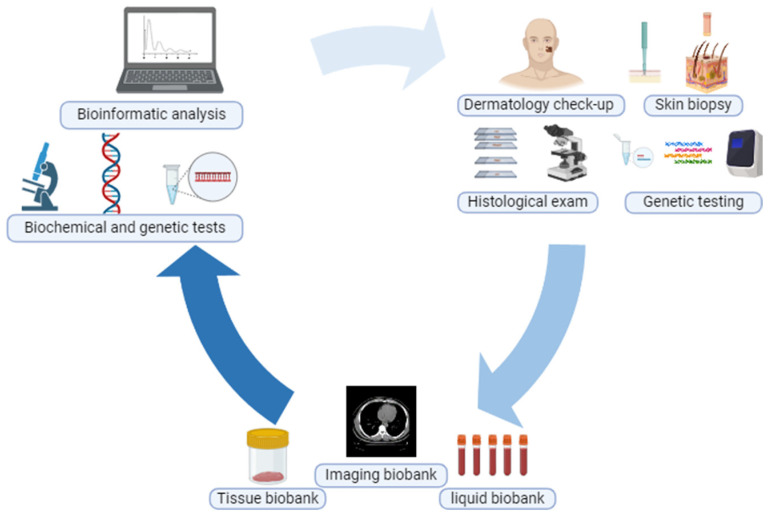
Schematic representation of melanoma patient diagnostic/clinical trial.

Melanoma represents only a small percentage of skin cancers, but of all of them, it is the leading cause of death each year of an estimated number of people. It is a multifactorial disease in that environmental, behavioral and genetic factors contribute to its onset [11]. Genetically, melanoma is fairly well-defined, and although the mutations that fuel it can be many, the major ones (also in terms of percentage of cases) are those affecting the genes necessary for cell proliferation, such as BRAF and NRAS [10,52]. In recent years, it has been one of the cancers that has greatly benefited, in terms of survival, from the advent of target therapies and immunotherapy. Nevertheless, many aspects about its genesis, progression and development of resistance to treatments remain unclear. Moreover, its high biological complexity (also related to the close interconnections it establishes with its microenvironment) makes the study of melanoma still very active and proliferative [11]. Given its highly heterogeneous characteristics, melanoma still emerges as an open challenge in the development of new therapies and the discovery of new biomarkers. Indeed, despite the progress made, still too many patients suffer disease progression or do not respond to treatments in any way [53,54]. An important aspect on which the research is focusing, not only for melanoma but also for other skin cancers, concerns the study of tumor heterogeneity between different patients. Simply, not all melanoma cases with similar characteristics respond in the same manner to treatments, further highlighting the need for personalized medicine structured on a single clinical case [55] (Figure 4).

Therefore, it is clear that biobanking and the integration of data from different sources are invaluable sources to draw on for the understanding of still obscure melanoma aspects. Although not yet as developed as in the case of other pathologies, melanoma biobanks are slowly emerging as new realities. As previously mentioned, compared with other diseases, melanoma-oriented biobanks are still in the embryonic and defining stages. A recent and interesting prospective study, called BioMel, has set the basis for the creation of a melanoma biobank in which biological samples, epidemiological information and medical data of patients with primary and advanced melanoma or equivocal lesions are collected (https://biomel.org, accessed on 1 July 2024). The primary aim of this project is to improve diagnostics, prognostics and therapy outcomes of melanoma patients. BioMel represents an almost unique biobanking reality, as at the time of its establishment, there were no other biobanks in Europe able to collect and catalog tissue and blood samples from melanoma patients in a standardized manner [56]. An important role in melanoma biobanking is played by the Melanoma Institute Australia (MIA) Biospecimen Bank, the largest collection of melanoma tissue samples from 10,000 patients (https://melanoma.org.au, accessed on 1 July 2024). This rapidly developing entity has enabled the development of numerous projects for the analysis and research of new prognostic markers in melanoma [57,58,59]. In addition to these two established entities, other melanoma biobanks are emerging in recent years, indicating the importance of a more extensive and large-scale study for this disease. One example is the Cancer Moonshot Biobank-Melanoma Collection (https://moonshotbiobank.cancer.gov, accessed on 1 July 2024), established in 2016, which collects healthy and tumor samples from melanoma patients. Recently, a study based on the use of these samples highlighted the high genomic and transcriptomic complexity of melanoma and the need for personalized treatment [60].

Melanoma follow-up involves the periodic evaluation of radiological investigations of various types. So, for this pathology, the amount of imaging data available is extremely significant, and several studies have shown that the image analysis makes it possible to obtain important information about prognosis and treatment response [37,61,62,63]. Although it is clear that this approach can help improve the study and comprehension of melanoma, including by identifying new imaging biomarkers, dedicated biobanks for this pathology are not yet available. This appears to be a great limitation because the imaging could be integrated with the biological data already available, creating study platforms with high scientific relevance.

## 6. Future Perspective and Conclusions

The last few decades revolutionized the concept of medicine and therapy, no more seen in their generality but associated with the individual. The ability to draw from endless sources of biological and imaging data is linked to sensitive biobanking processes. Although the way is promising, much still needs to be implemented in order to achieve 100% functional biobanks. The starting point will be to reorganize less-structured biobanks, such as imaging biobanks, by implementing them with genomic and clinical data, exploiting, where possible, AI and machine learning to identify new biomarkers and, thus, actively contribute to precision medicine. One of the weaknesses, indeed, is the lack of interconnection and communication between the various players involved in biobanking. A major effort will be the development of secure, open-access platforms for accessing biobanked data, so that the information can be accessed by more researchers either from within or outside the target institution but in complete privacy and security. In an increasingly digital era, the safeguarding of individual data, indeed, must remain a solid point in the governance of biobanks through defined legislation.

The economic aspect will be no less important in the future. The management of billions of data is expensive and constantly requires economic sources to draw on. Biobanks have undefined costs that can evolve over time. Indeed, even depending on their objective, they are expensive in terms of personnel, equipment, consumables, etc. [64]. To date, biobanks are not able to fully meet their costs but must rely on funding linked to research projects or donations. Support from local public authorities would also be desirable for the future. The implementation of business plan systems, the creation of public–private collaborations and, especially, long-term funding policies would ensure greater input into scientific research. In addition, introducing AI into biobank management could optimize workflows and reduce operational costs.

## Data Availability

Not applicable.
